# Effect of IIFAR information nursing model and comprehensive rehabilitation training on negative emotions and rehabilitation outcomes in elderly hemiplegic stroke patients

**DOI:** 10.4314/ahs.v25i1.23

**Published:** 2025-03

**Authors:** Yinli Duo, Guiping Zhong, Zequn Shen

**Affiliations:** 1 Geriatric and Special Needs Medicine Department of Surgical Ward, General Hospital of Ningxia Medical University, Yinchuan, China; 2 Disinfecting Supply Room, The Second Affiliated Hospital of Hainan Medical College, Haikou, China; 3 School of Nursing, Hangzhou Vocational and Technical College, Hangzhou, China

**Keywords:** IIFAR information nursing, comprehensive rehabilitation training, elderly, stroke with hemiplegia, negative emotions

## Abstract

**Background:**

We aimed to investigate the effects of the IIFAR (initial check, information exchange, final accuracy check, reaction) nursing model and comprehensive rehabilitation training on negative emotions and rehabilitation outcomes in elderly stroke patients with hemiplegia.

**Methodology:**

Forty elderly stroke patients with hemiplegia were divided into two groups: the control group received comprehensive rehabilitation training intervention, while the observation group received IIFAR information nursing model intervention. Both groups were assessed for disease uncertainty, negative emotions, mindfulness level, balance, and limb movement ability before and after the intervention.

**Results:**

After the intervention, both groups experienced a significant decrease in the complexity, ambiguity, lack of information, unpredictability, State Anxiety Inventory, Trait Anxiety Inventory, and total score of the State-Trait Anxiety Inventory scale. However, the observation group had lower scores than the control group. Furthermore, after the intervention, both groups saw a significant increase in scores, but the observation group had higher scores than the control group. Lastly, after the intervention, the observation group had significantly higher scores than the control group.

**Conclusion:**

The use of the IIFAR information nursing model combined with comprehensive rehabilitation training can improve the disease uncertainty and negative emotions of elderly stroke patients with hemiplegia, and enhance their mindfulness level and balance movement ability.

## Introduction

Stroke can cause cognitive and motor dysfunction, and has a high disability and mortality rate^1^. Hemiplegia is a common sequela, with patients experiencing limb spasm, poor mobility, and balance abnormalities. For some patients, especially elderly individuals with weaker physical function, negative emotions such as anxiety and despair can be triggered due to long-term illness and heavy economic burdens. These negative emotions can reduce patients' adaptability and treatment compliance, thereby affecting treatment outcomes. Hospital health education is an important way to increase patient disease awareness, correct wrong behaviors, and maintain physical and mental health^2^. The IIFAR information nursing model can complete interactive education with patients through four processes, including initial verification and information exchange, ensuring the rationality and effectiveness of the provided information, and relieving patients' stress and anxiety^3^. Currently, this nursing model plays an important role in the perioperative care of various patients^3^, but there are few reports on its intervention effect on hemiplegic stroke patients. This study selected 80 elderly patients as the research subjects to guide clinical intervention and obtained the following report.

## Patients and methods

### Patients

Using the implementation time of our hospital's IIFAR information nursing model (February 2022) as the boundary, 80 elderly hemiplegic stroke patients who visited our hospital before the implementation (June 2021 to January 2022) were selected as the control group and received comprehensive rehabilitation training intervention. Forty patients who visited the hospital after the implementation (February 2022 to October 2022) were selected as the observation group and received IIFAR information nursing model intervention based on the control group. Inclusion criteria: 1) meet the diagnostic criteria for ischemic stroke^4^; 2) aged 60-85 years old, first onset, unilateral hemiplegic lesion; 3) stable condition, no cognitive impairment, mini-mental state examination (MMSE)^5^,^6^ >27 points; 4) voluntary participation in the study, able to cooperate with the study independently or with the accompaniment of family members. Exclusion criteria: 1) those with joint arthritis, limb deformities, or other conditions affecting balance and motor function; 2) those with acute or chronic infections, respiratory failure, cognitive impairment, or malignant tumors; 3) those with significant organ dysfunction; 4) those with mental abnormalities or recurrent stroke. The patients included in the study had no previous history of stroke and had been diagnosed with ischemic stroke based on the diagnostic criteria established by the World Health Organization (WHO) in 2018. The patients had also undergone a comprehensive medical evaluation to ensure that they met the study's inclusion criteria. The general information of the two groups was comparable (P>0.05), as shown in Table 1. The study was approved by the hospital ethics committee, which evaluated the study's ethical implications and ensured that all patient data were kept confidential and anonymous. The patients were informed about the study's purpose and procedures and provided written consent before participating in the study.

**Table 1:** Comparison of general data between the two groups [x± s, n (%)]

Group	n	Gender		Age	Disease duration	Hemiplegic side		Combined	Combined
		Male	Female	(years)	(Months)	LEFT	Right	hypertension	diabetes
Observation group	40	21 (52.50)	19 (47.50)	72.35 ± 6.27	3.21 ± 0.79	23 (57.50)	17 (42.50)	9 (22.50)	6 (15.00)
Control group	40	25 (62.50)	15 (37.50)	73.02 ± 7.10	3.59 ± 0.81	19 (47.50)	21 (52.50)	11 (27.50)	4 (10.00)
t/*χ*^2^ value			0.818	0.447		0.802		0.267	0.457
P-value			0.366	0.656		0.370		0.606	0.499

## Methods

The control group received comprehensive rehabilitation training intervention, which consisted of physical therapy, occupational therapy, and speech therapy, as well as educational and psychological support. The rehabilitation training was conducted by a team of experienced therapists and tailored to the individual needs of each patient. The training sessions were conducted on a regular basis for a period of six months, and the patients were encouraged to practice the exercises at home. The comprehensive rehabilitation training intervention included limb function training, daily living ability training, swallowing training, and breathing training. For limb function training, when patients were unable to turn over by themselves, passive limb movements were performed by fixing one hand near the proximal joint and placing the other hand near the distal joint for gentle rhythmic movement, helping the shoulder joint, knee joint, and other joints to stretch. Limb massage was also conducted twice a day, gradually increasing in intensity and depth. When patients were able to turn over by themselves, sitting training was performed by sitting on the bed with a table in front of them and a pillow behind their back. The affected side knee joint was flexed at 90 degrees, and the weight was placed on the hips. Standing training was conducted when the patient could stably grasp the sitting training, with support from two people on both sides, and then progressed to balancing exercises, such as reaching for objects and sitting-to-standing balance training.

The above training was conducted twice a day for 20-30 minutes each time. Daily living ability training involved practicing tasks such as eating with utensils, using the toilet, showering, and dressing. Patients were assisted as needed and instructed on proper technique and safety considerations. Swallowing training involved cleaning the patient's oral cavity with a frozen cotton swab to stimulate the tongue root muscle group, followed by saliva swallowing training after deep breathing and holding their breath for 3 seconds. Breathing training included pursed lip breathing and abdominal breathing, with each training performed twice a day.

The observation group received IIFAR information nursing model combined with comprehensive rehabilitation training intervention, similar to the control group. Four sessions of IIFAR information nursing were conducted on the 1st, 3rd, and 7th days of hospitalization and the day before discharge. The nursing topics were as follows: stroke-related health knowledge and diet, medication nursing, negative emotion regulation methods, rehabilitation training and prevention of complications nursing, and home care priorities after discharge. Each nursing session lasted about 45 minutes, with specific operating procedures shown in Table 2.

**Table 2:** Operating Process of IIFAR Information Care Mode

Step	Nursing Contents
Initial Reconciliation	1. Assess whether patients have negative emotions and cognitive impairments. 2. Confirm whether patients are in the best state to receive information and determine whether they have an information need. 3. Communicate with patients and guide them to describe their current knowledge of stroke-related information such as medication, diet, and activity, in order to determine the patient's level of disease understanding. 4. Basedon the patient's description, determine the specific information needed.
Information exchange	To provide elderly hemiplegic stroke patients and their families with the necessary information regarding medication, nursing precautions, and prevention of complications, a relevant information package will be created. This package will be delivered to the patients and their families using various formats such as PPT presentations and videos. The number of information packages will not exceed five and will be followed by a question and discussion session lasting approximately 5-10 minutes. The families are encouraged to record the information delivery process to enhance their memory retention.
Final Accuracy Check	1. During patient and family interactions, kindly guide them to repeat the key points of information exchanged using their own language. 2. Evaluate the patient and family's level of disease-related knowledge, point out any deficiencies, and provide additional education on correcting misinformation and filling knowledge gaps.
Reaction	1. Patients and family members should rest for 1-3 minutes and maintain a relaxed state. 2. Nursing staff should discuss with patients and their families the gains and feelings from the information conveyed during this session.

### Observation Indicators

Disease Uncertainty: The Disease Uncertainty Scale developed by Michei^7^ was used to measure the complexity, ambiguity, information inadequacy, and unpredictability of hospitalized patients. It consists of 33 items rated on a 5-point Likert scale, with the 15th item not scored. The total score ranges from 32 to 160, with higher scores indicating greater uncertainty.

Negative Emotions: The State-Trait Anxiety Inventory (STAI)^8^ was used to measure anxiety, including the State Anxiety Inventory (S-AI) and Trait Anxiety Inventory (T-AI), with a total of 40 items rated on a 0-4 scale. Higher scores indicate greater anxiety.

Mindfulness Level: The Mindful Attention Awareness Scale (MAAS)^9^ was used to measure mindfulness, consisting of one dimension and 15 items rated on a 6-point Likert scale. The total score ranges from 16 to 90, with higher scores indicating higher mindfulness levels.

Balance Ability: The Berg Balance Scale (BBS)^10^ was used to assess balance ability, consisting of 14 items rated on a 5-point Likert scale. The total score ranges from 0 to 56, with higher scores indicating better balance ability.

Limb Motor Function: The Fugl-Meyer Motor Function Scale (FM)^11^ was used to assess upper limb motor function (66 points) and lower limb motor function (34 points), with a total score of 100. Higher scores indicate better limb motor function.

### Statistical Analysis

Statistical Product and Service Solutions (SPSS) 22.0 (IBM, Armonk, NY, USA) was used for data analysis. The measurement data of disease uncertainty and negative emotions in the two groups were expressed as mean ± standard deviation and analyzed using t-tests. The count data of gender and hemiplegic side were expressed as n (%) and analyzed using chi-square tests. Differences with P<0.05 were considered statistically significant.

## Results

### Comparison of disease uncertainty between the two groups

Before intervention, there was no statistically significant difference in Michei's disease uncertainty scale complexity, uncertainty, information deficiency and unpredictability scores before intervention between the observation group and the control group (P > 0.05). At discharge, the observation group and the control group all showed a significant decrease in uncertainty scores, with the observation group showing a greater reduction than the control group (P < 0.05), as shown in Table 3.

**Table 3:** Comparison of disease uncertainty between the two groups (x ± s, points)

Group	n	Ambiguity		Complexity		Information lacking		Unpredictability	
		Pre-intervention	At discharge	Pre-intervention	At discharge	Pre-intervention	At discharge	Pre-intervention	At discharge
Observation group	40	50.12 ± 5.27	20.26 ± 4.75*	36.33 ± 4.19	20.51 ± 3.02*	20.14 ± 3.02	10.12 ± 2.30*	19.13 ± 2.62	11.33 ± 1.18*
Control group	40	50.08 ± 5.05	31.08 ± 4.33*	35.10 ± 4.01	25.07 ± 3.64*	20.20 ± 3.13	13.31 ± 2.19*	19.20 ± 2.80	13.67 ± 1.25*
t-value		0.035	10.647	1.341	6.098	0.087	6.353	0.115	8.609
P-value		0.972	< 0.001	0.184	< 0.001	0.931	< 0.001	0.908	< 0.001

Note: Compared with that before intervention in the same group

* *P* < 0.05

### Comparison of negative emotions between the two groups

There was no statistically significant difference in S-AI score, T-AI score and total score of STAI scale before intervention between the two groups (P > 0.05). At discharge, both groups showed a significant decrease in negative emotion scores, with the observation group showing a greater reduction than the control group (P < 0.05), as shown in Table 4.

**Table 4:** Comparison of negative emotions between the two groups (x̅ ± s, points)

Group	n		S-AI	T-AI		STAI total score	
		Pre-intervention	At discharge	Pre-intervention	At discharge	Pre-intervention	At discharge
Observation group	40	58.21 ± 5.76	45.31 ± 4.27*	56.33 ± 5.21	40.26 ± 4.19*	114.54 ± 16.35	85.57 ± 10.12*
Control group	40	58.02 ± 6.10	50.25 ± 4.58*	56.79 ± 5.14	43.37 ± 4.02*	114.81 ± 15.60	93.62 ± 10.11*
*t* value		0.143	4.990	0.398	3.387	0.076	3.559
*P* value		0.887	< 0.001	0.692	< 0.001	0.940	< 0.001

Note: Compared with that before intervention in the same group

* *P* < 0.05

### Comparison of mindfulness level between the two groups

Before intervention, the MAAS scale score before intervention was (52.63 ± 6.10) points in the observation group and (53.08 ± 7.56) points in the control group, and there was no systematic difference between the two groups (t = 0.293, P = 0.770). At discharge, the mean score in the observation group was significantly higher than that in the control group (75.88 ± 9.25 vs. 70.08 ± 8.72, t = 2.886, P = 0.005).

### Comparison of motor balance ability between the two groups

Before intervention, there was no statistically significant difference in FM upper limb and lower limb motor function scores and BBS scale scores before intervention between the two groups (P > 0.05). At discharge, both groups showed a significant improvement in motor function, with the observation group showing a greater improvement than the control group (P < 0.05), as shown in Table 5.

**Table 5:** Comparison of motor balance ability between the two groups (x̅ ± s, points)

Group	n	FM Upper Extremity Motor Function		FM lower limb motor function		BBS Scale	
		Pre-intervention	At discharge	Pre-intervention	At discharge	Pre-intervention	At discharge
Observation group	40	32.14 ± 4.19	57.24 ± 4.02^*^	20.02 ±3.51	26.77 ± 4.08^*^	40.13 ± 3.02	50.26 ± 5.09
Control group	40	32.89 ± 4.06	53.05 ± 4.16^*^	19.90 ±3.77	22.39 ± 4.15^*^	39.90 ± 3.64	46.71 ± 5.02
t-value		0.813	4.581	0.147	4.760	0.308	3.141
P-value		0.419	< 0.001	0.883	< 0.001	0.759	0.002

Note: Compared with that before intervention in the same group

* P < 0.05

## Discussion

Due to central nervous system damage, sensory impairment, decreased core muscle control ability, and reduced limb flexibility, hemiplegic stroke patients experience a significant impact on their quality of life^12^. Scientific and reasonable rehabilitation training has been shown to promote patient recovery and improve prognosis. Comprehensive rehabilitation training combines various training content, such as limb function training, daily living ability training, and swallowing training, and progresses from easy to difficult, which can help patients recover neurological function, muscle strength, and limb sensation^13^.

However, most elderly patients are vulnerable to negative emotions such as depression during the treatment process, which may result in poor treatment compliance and hinder post-disease recovery. This study shows that the IIFAR information nursing model intervention can compensate for the shortcomings of single comprehensive rehabilitation training, and can effectively improve patient disease uncertainty and negative emotions, as well as increase mindfulness and limb movement function.

The study found that the dimensional scores of the Michei disease uncertainty scale in the observation group were significantly lower than those in the control group at discharge, thanks to the professional and systematic education and nursing provided by the IIFAR information nursing model. Patients' disease uncertainty mainly arises from a lack of disease-related knowledge and uncertainty about their own disease progression^14^. Hemiplegic stroke patients in the elderly population often have decreased memory and poor information acceptance abilities, and conventional health education only provides standardized education content, neglecting individual factors, which leads to less-than-ideal education results.

In the IIFAR information nursing model, the initial verification stage evaluates the patient's emotional status and knowledge acceptance status, analyzes the required information content through communication with the patient, and facilitates targeted nursing. Subsequent information exchange divides the necessary education content into multiple information packags and uses various forms of explanation, such as videos and PPTs, to reduce memory difficulty, in accordance with human memory rules. The final accuracy verification stage helps patients review the education content, correct erroneous memory, and consolidate the education effect. IIFAR information nursing model can reduce disease uncertainty in families of children with neuroblastoma and improve treatment satisfaction, providing theoretical support for this study.

According to our study, the total score of the STAI scale for the observation group at discharge was lower than that of the control group, while the MAAS scale score was higher than that of the control group. This suggests that the IIFAR information nursing model combined with comprehensive rehabilitation training can alleviate negative emotions such as anxiety and worry and improve mindfulness levels compared to single comprehensive rehabilitation training. Our analysis suggests that the high-quality health education provided by the IIFAR information nursing model can address patients' specific information needs, such as medication guidance and complication prevention, which can improve disease awareness and control, and appropriately release anxiety. Additionally, in our study, family members were required to participate in each information nursing session, and nursing staff and family members paid attention to the psychological and physiological needs of patients, providing more emotional comfort and social support^15^.

Furthermore, our study found that the balance function of the observation group at discharge was significantly improved compared to the control group. This may be due to the four information nursing sessions that patients received after admission, which focused on stroke-related knowledge, diet, rehabilitation training, and complication prevention. These sessions helped to alleviate negative emotions, correct misconceptions, standardize rehabilitation training movements, improve training enthusiasm, and promote rehabilitation. In conclusion, providing elderly hemiplegic stroke patients with the IIFAR information nursing model combined with comprehensive rehabilitation training can improve disease uncertainty and negative emotions, increase mindfulness levels and balance motor ability, and has a good clinical application basis.
